# Hepatic Transcriptomics of Broilers with Low and High Feed Conversion in Response to Caloric Restriction

**DOI:** 10.3390/metabo14110625

**Published:** 2024-11-14

**Authors:** Adewunmi O. Omotoso, Henry Reyer, Michael Oster, Siriluck Ponsuksili, Barbara Metzler-Zebeli, Klaus Wimmers

**Affiliations:** 1 Research Institute for Farm Animal Biology (FBN), 18196 Dummerstorf, Germany; omotoso@fbn-dummerstorf.de (A.O.O.); reyer@fbn-dummerstorf.de (H.R.); oster@fbn-dummerstorf.de (M.O.); ponsuksili@fbn-dummerstorf.de (S.P.); 2 Centre for Veterinary Systems Transformation and Sustainability, Clinical Department for Farm Animals and Food System Science, University of Veterinary Medicine Vienna, 1210 Vienna, Austria; barbara.metzler@vetmeduni.ac.at; 3 Faculty of Agricultural and Environmental Sciences, University Rostock, 18059 Rostock, Germany

**Keywords:** meat-type chicken, resource allocation, restrictive feeding, mRNA profiling, hepatic metabolism

## Abstract

Background: In broiler chickens, the efficient utilization of macro- and micronutrients is influenced by various metabolic pathways that are closely linked to feed efficiency (FE), a critical metric in poultry industry, with residual feed intake (RFI) as the preferred proxy. Feed restriction is considered an approach to address the underlying molecular mechanisms of feed conversion. We hypothesized that broiler chickens with divergent RFI subjected to quantitative feed restriction differ in their pattern of molecular pathways for efficient nutrient utilization in liver as post-absorptive tissue. Methods: Cobb 500FF broiler chickens divergent for RFI (*n* = 112) were feed-restricted from day 9 until market weight at day 33–37 post-hatch. Based on a previous trial, feed restriction levels were set at 92% (low-RFI birds) and 80% (high-RFI birds) relative to the control groups. Transcriptomic analyses of the liver were conducted. Results: Due to the interaction of the RFI group and feeding regimen, a total of 140 to 507 differentially expressed genes were identified for the respective contrasts, with implications for hepatic metabolism and cellular stress response. Although the broilers did not realize their full growth potential under restrictive feeding (12.4% reduced body weight vs. controls, *p* = 0.094), the gene expression patterns indicate a lower susceptibility to blood coagulation (*KNG1*, *FGG*, and *FGB*), suggesting that controlled and mild feed restriction could lead to health benefits in less feed-efficient broilers. Moreover, FE traits are shown to be linked to cellular detoxification processes (*MGST3* and *CYP2AC2*) and triacylglycerol syntheses (*MOGAT1* and LPIN1). Conclusions: Divergent transcriptional profiles between broiler groups under varied caloric conditions indicate potential for optimizing nutritional management strategies.

## 1. Introduction

Feed efficiency (FE), a multifaceted quantitative trait, is a critical metric in the poultry industry and generally expressed as the feed conversion ratio (FCR), which is the ratio of feed intake (FI) to body weight gain (BWG) over a specific period. To account for the bird’s capacity to utilise feed for both maintenance and performance, the residual feed intake (RFI) has been implemented [1,2]. The RFI approach minimises interference from the host-specific confounding factors, including variations in age, body size, sex, and genetics [3]. In fact, the selection towards improved FE in monogastric species emphasises the significance of RFI as the preferred proxy [4,5].

Recent studies highlighted a number of factors which impact on FE, such as host genetics showing moderate heritability in chickens [6,7], management practices including hygiene regimen and disease prevention [8,9,10], and nutritional factors to ensure digestibility and maintain intestinal health [11,12]. Due to the ecological concerns and economic demands associated with animal husbandry, selection strategies for FE in broiler chickens are continuously scrutinised [13,14], but it has been suggested that a further reduction in environmental impact is becoming impractical for the broiler sector in conventional management systems [15].

Using transcriptomics, metabolomics, and microbiomics, molecular features associated with high FE in broiler chickens provided insight into physiological mechanisms mediated by different host compartments, such as small intestine and breast muscle [16], serum metabolites [14,17], and gut/faecal microbiota [18]. The transcriptional pattern between broiler chickens with divergent FE revealed an enhancement in FXR and RXR molecular pathways and mitochondrial function in feed-efficient broilers as a potential mechanism for promoting resource utilisation and nutrient allocation [16,19]. In addition, a number of other biological processes, such as lipid utilisation, bile salt transport, and ketogenesis in the intestinal tissue and muscles, are assumed to affect FE.

Controlled feed restriction can be an effective tool for improving FE [20]. Practical applications of feed restriction on broiler farms may be limited, but strategies like time-restricted feeding, behavioural interventions, and nutritional modifications (e.g., nutrient density and crude protein levels) offer promising opportunities for implementation [21,22]. To gain insight into metabolic pathways and trade-offs due to the significantly reduced FI in commercial poultry flocks, broiler chickens were fed 5–10% less feed compared to ad libitum counterparts in recent studies [14,23]. Analyses revealed a number of serum metabolites with higher (e.g., asparagine and citrulline) or lower levels (e.g., taurine and uric acids) due to feed restriction in broiler chickens pointing to responses of the hepatic metabolism and nutrient utilisation. These disparities in serum metabolite profiles between ad libitum and restrictively fed birds were further linked with alterations in the hepatic fatty acid synthesis and variations in energy metabolism for gluconeogenesis [14]. The metabolic pathways affected by feed restriction in chickens could further reveal targets for pharmaceutical interventions to address body weight status and metabolic health status in other species, including humans.

To elucidate the molecular mechanisms underlying the effects of high and low RFI in broilers on ad libitum and restrictive feeding, this study employs comprehensive transcriptomic analyses of the liver. Given the crucial role of the liver in processing and redistribution of metabolic fuels, endocrine control, and cellular stress responses, the investigations aim to identify differentially expressed genes (DEGs) and draw conclusions about the molecular signalling pathways involved. In addition, by comparing birds with high and low RFI, differences in coping strategies used by the liver in response to reduced feed intake are analysed.

## 2. Materials and Methods

Ethical Statement: To ensure the handling and treatment of birds according to ethical standards, all procedures comprised in this study were approved by the ethics committee of the University of Veterinary Medicine Vienna and the Austrian national authority, according to paragraph 26 of the Law for Animal Experiments, Tierversuchsgesetz 2012-TVG 2012 (experimental protocol number: GZ 68.205/0148-II/3b/2015).

### 2.1. Experimental Animals, Housing, and Tissue Sample Collection

A total of 112 day-old Cobb 500FF broiler chickens (55 males and 57 females) were procured from a commercial hatchery (Brüterei Schlierbach GmbH, Pettenbach, Austria). The animal trial consisted of two independent batches, with 56 birds per batch. Experimental housing and feeding regimens have been elaborately documented [14,18]. Briefly, birds were housed in groups of four for the first week before being transferred to individual metabolic units to determine the individual feed intake of each bird. Daily health checks were conducted in compliance with animal welfare standards. The dietary composition was based on a standard corn–soybean formulation provided to the birds throughout their different productive stages (starter, days 1–8, crude protein (CP): 228 g/kg DM, metabolizable energy (ME): 12.0 MJ/kg DM; grower, days 9–20, CP: 207 g/kg DM, ME: 12.0 MJ/kg DM; and finisher, days 21–37, CP: 194 g/kg DM, ME: 12.4 MJ/kg DM; Supplementary Table S1). Depending on the dietary regime adopted for the two feeding groups, the feed ration on day 9 was administered either ad libitum or quantitatively restricted. The restriction of the feed quantity was based on a previous study with the same genetics and corresponded to 90–95% of the experienced ad libitum feed consumption [24]. Due to the restrictive feeding approach, the amount of starch and CP in the diet was reduced equally. Birds had unrestricted access to demineralized water throughout the experiment. Zootechnical parameters comprising the individual daily feed intake (FI) and body weight (BW) were recorded weekly on days 1, 7, 14, 21, 28, and the respective slaughtering day (between days 33 and 37 post-hatch). Additionally, the metabolizable mid BW and the feed conversion ratio (FCR) were calculated. The determination of FE and the calculation of RFI of the experimental birds has already been previously described in detail [14,18]. In brief, a non-linear mixed model was employed to estimate the RFI using total FI, body weight gain (BWG), and mid-test metabolic body weight obtained between days 7 and 30. From each experimental batch and sex, birds with the lowest RFI (indicating high FE) and with the highest RFI (indicating low FE) were selected for slaughter and tissue sampling. Birds were euthanized between days 33 and 37 post-hatch using a lethal dose of sodium thiopental (50–100 mg/kg, medicamentum pharma GmbH, Allerheiligen im Mürztal, Austria) administered intravenously. After sacrificing the birds, the right liver lobe was obtained, cut into pieces, snap-frozen in liquid nitrogen, and stored at −80 °C until analysis. Liver samples from 32 birds balanced for batch, sex, FE-group, and feeding regimen were used for subsequent RNA isolation. Individual BW measurements and RFI values were compared for selected birds using a linear mixed model (R language v4.2.2; R package lmerTest, v3.1-3; R foundation for statistical computing, Vienna, Austria). The model included RFI and sex, as well as their interaction, as fixed effects and the day of slaughter as a random effect. The *p*-values were adjusted for multiple comparisons using the Tukey–Kramer procedure (R, lsmeans package, v2.30-0). The significance level was set at *p* ≤ 0.05.

### 2.2. RNA Isolation and Purification

The protocol for the extraction of total RNA included an initial extraction with TRI reagent (Sigma-Aldrich, Taufkirchen, Germany), followed by treatment with DNase I (Roche, Mannheim, Germany) and purification of the RNA with the NucleoSpin RNA II column-based extraction kit (Macherey-Nagel, Düren, Germany). The extracted RNA was checked for quantity and quality using the NanoDrop ND-1000 photospectrometer (NanoDrop, Peqlab, Erlangen, Germany) and a nucleic acids separation on a denaturing agarose gel electrophoresis to assess RNA integrity. Furthermore, to ensure the absence of genomic DNA contamination, RNA samples were amplified using a polymerase chain reaction (PCR) targeting the chicken GAPDH gene (intron-spanning primers: forward primer: 5′-AGTCGGAGTCAACGGATTTG-3′; reverse primer: 5′-CTGCCCATTTGATGTTGCTG-3′).

### 2.3. Microarray Processing and Data Analysis

Liver samples were collected and analysed using holistic transcriptomic profiling. Therefore, total RNA was converted into biotin-labelled and fragmented single-stranded cDNA using the WT Plus Expression Kit (Affymetrix, Santa Clara, CA, USA) and hybridised to Chicken GeneChip Gene 1.0 ST arrays (Affymetrix). The array was processed as described in the manufacturer’s instructions using the GeneChip hybridisation, washing, and staining kit (Affymetrix). After scanning the chips, the raw intensity values were captured using the Affymetrix GCOS 1.1.1 software and normalised with the Robust Multichip Average algorithm of the affy R package. Data quality was assessed using the R package arrayQualityMetrics (v3.54.0), taking into account three factors: distance between samples, signal intensity distribution, and individual data set quality. One microarray that failed in two of the categories was excluded from the study (group: restrictive diet, high RFI, female). A pre-filtering to exclude probe sets with low means and high standard deviation (mean: lower 5% quantile for all samples; standard deviation: upper 5% quantile within each group), control probe sets, and internal controls was performed to improve subsequent statistical analyses. The probe set annotation was updated according to the Ensembl GRCg6a reference genome (release 106) [25].

Differential expression was analysed in the R environment using the linear models for microarray analysis (limma) package (v3.54.2). Residuals of expression values were used after removing the effects of the experimental run (two runs), the laboratory batch of cDNA generation (four batches), and the gender of the chickens. The subsequent linear model included the interaction of RFI class (high FE, low FE) and feeding regimen (ad libitum, restricted) as a fixed effect. Fold changes, which reflect differences in expression between the different groups, were calculated from the least square means. Probe sets with a *p*-value < 0.01 in the statistical analysis were considered significant, and the corresponding differentially expressed genes (DEGs) were derived from the gene annotation. Enrichment analyses were performed on the DEGs in reference to the Gene Ontology terms—Biological Processes, KEGG pathways and the Reactome database with g:profiler (https://biit.cs.ut.ee/gprofiler_archive3/e106_eg53_p16/gost, accessed on 26 March 2024).

## 3. Results

Regarding high-RFI birds, body weight was significantly lower in feed-restricted birds compared to their ad libitum counterparts (Table 1). The metabolizable mid BW was unaffected by RFI group but lowered due to the applied feed restriction. For RFI and FCR values, there were design-related significant differences between the low- and high-RFI groups regardless of the feeding regime. The ad libitum-fed birds with low FE had significantly higher RFI values than the restricted-fed birds with low FE. The mortality rates were inconspicuous in all experimental groups (<5%). The effects on RFI for the subset of birds selected for the transcriptome analysis were in agreement with the overall experiment [18].

**Figure 1:**
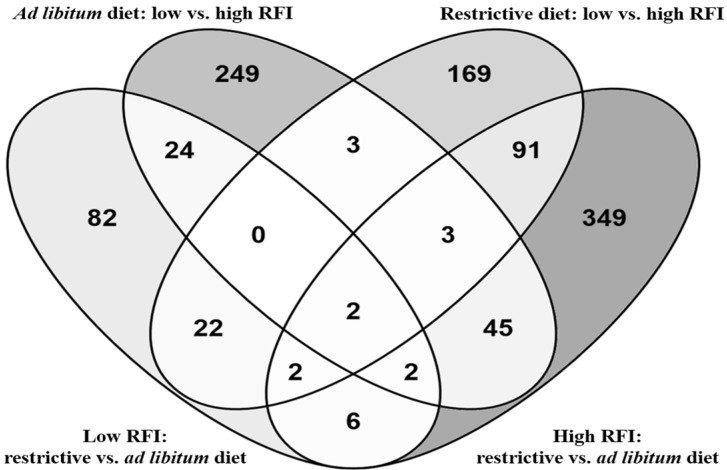
Venn diagram of differentially abundant liver probe sets (*p* < 0.01) resulting from the comparison of low- and high-RFI broiler chickens subjected to an ad libitum vs. a restrictive feeding regimen. The values indicate the number of significant probe sets in the respective comparisons. Overlaps refer to commonly regulated DEGs between multiple comparisons.

The analysis of differential expression of hepatic transcripts in the interaction of the RFI group and feeding regimen revealed 140 to 507 differentially abundant probe sets (*p* < 0.01) in the respective contrasts (Figure 1). The comparison of the high-RFI birds under ad libitum and restrictive feeding resulted in the largest number of differentially abundant probe sets.

**Figure 2:**
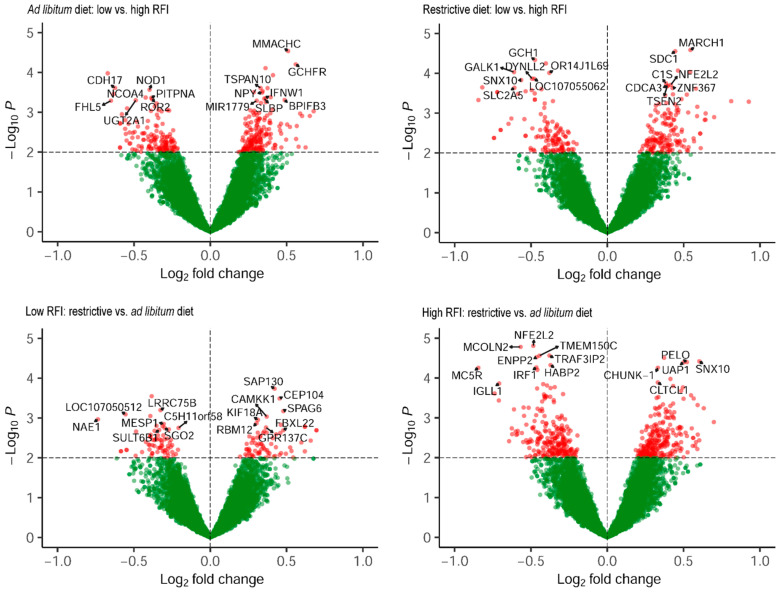
Volcano plots of genes expressed in the liver of low- and high-RFI broiler chickens subjected to an ad libitum vs. a restrictive feeding regimen. Differentially expressed genes (DEGs) exceeding the significance threshold are highlighted in red. The annotated top DEGs, selected according to *p*-value in each of the comparisons, are labelled.

**Figure 3:**
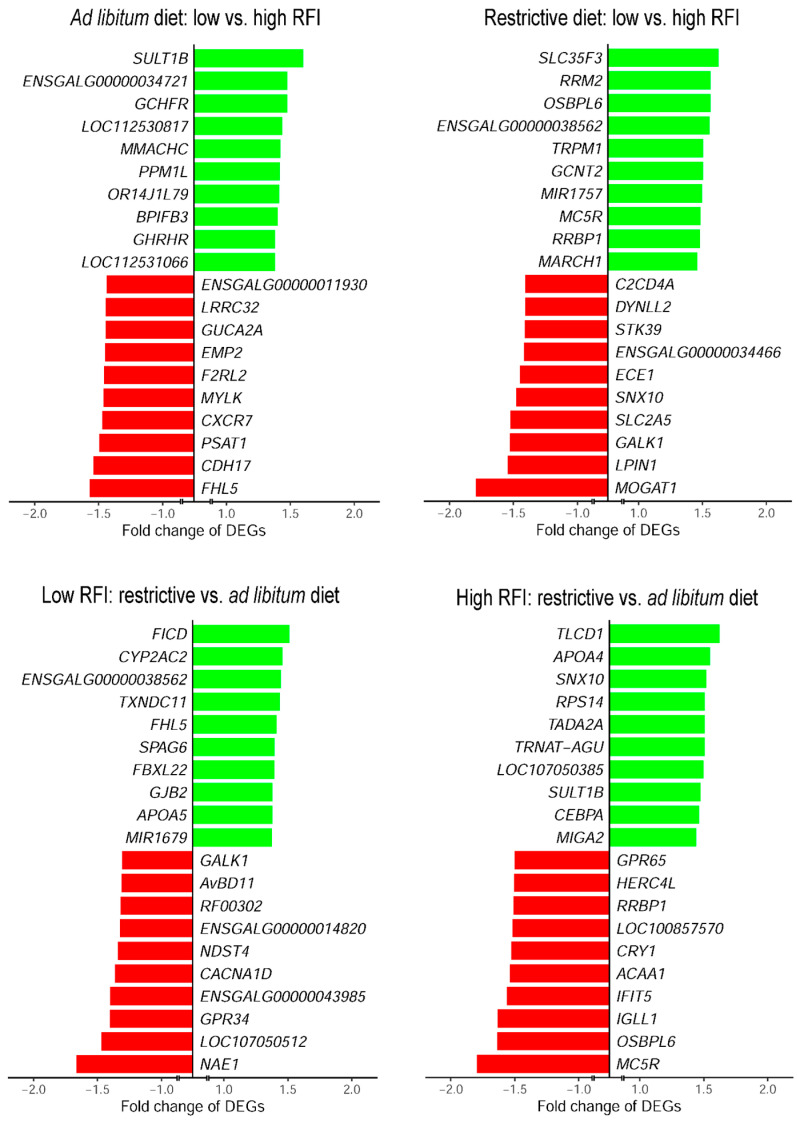
The top differentially expressed genes (DEGs) in the liver of low- and high-RFI broiler chickens subjected to an ad libitum vs. a restrictive feeding regimen. Each 10 upregulated (green) and 10 downregulated (red) transcripts were displayed per respective experimental comparison.

Based on the annotation of probe sets, the distribution of the data was visualised in volcano plots with the highest significant DEGs indicated (Figure 2). The identified most differentially abundant genes (fold change) are displayed in Figure 3.

In the ad libitum-fed birds, the genes *SULT1B*, ENSGALG00000034721, and *GCHFR* were the most strongly increased and *FHL5*, *CDH17*, and *PSAT1* were the most strongly decreased in their abundance in low-RFI compared to high-RFI broiler chickens (Figure 3). The applied feed restriction revealed the most increased mRNA abundances of *SLC35F3*, *RRM2*, and *OSBPL6*, and the most decreased mRNA abundances of *MOGAT1*, *LPIN1*, and *GALK1* in low-RFI compared to high-RFI birds. For low-RFI chicken, the comparison of the feeding regimen revealed the most increased expression of *FICD*, *CYP2AC2*, and ENSGALG00000038562 and the most decreased expression of *NAE1*, *LOC107050512*, and *GPR34* in birds which received the restricted diet compared to ad libitum-fed birds. The liver transcriptome profiling of high-RFI birds revealed *TLCD1*, *APOA4*, and *SNX10* to be the most upregulated and *MC5R*, *OSBPL6*, and *IGLL1* to be the most downregulated genes in response to restrictive feeding compared to ad libitum feed access.

The DEGs were subjected to enrichment analyses to gain insights into overarching biological functions and signalling pathways that are represented on the basis of gene sets (Table 2). The identified terms, although represented by only a few genes of the entire gene sets (Supplementary Table S2), indicate regulatory processes of liver integrity and homeostasis (e.g., cell–substrate adhesion, cell–cell adhesion, blood coagulation, and haemostasis) and provide indications for shifts in metabolism (e.g., superoxide metabolic process and drug metabolism—cytochrome P450).

## 4. Discussion

The high degree of efficient feed utilisation by broilers is a result of the continuous selection of genotypes over the last decades [26,27]. The magnitude of the difference in RFI between high- and low-RFI groups of the Cobb 500FF birds in the current study is similar to previous studies [4,16]. However, the reduction in body weight of the birds with high RFI and the fact that restrictive feeding showed an effect on total BWG in all birds of the trial [18] indicate that the broilers are unable to realise their full potential under restrictive feeding. Consistently, the metabolic mid BW as one of the indicators for the evaluation of FE was significantly reduced by the restricted feeding compared to the controls, indicating growth compensation of low-RFI birds throughout the trial period. The analysis of hepatic gene expression profiles in low- and high-FE chickens demonstrated significant alterations dependent on the feeding regimen with either ad libitum or restricted access to feed. However, transcriptomic data are useful to visualize the diversity of cellular and metabolic processes but represent only a snapshot. In addition, the dynamics of the endocrine loops via hepatic and/or gastrointestinal hormones cannot be taken into account.

### 4.1. Low RFI vs. High RFI Under Ad Libitum Feeding

High- and low-RFI birds achieved comparable growth rates, with the high-RFI group requiring a higher feed intake, highlighting their inherent differences in FE in agreement with previous studies [16,28]. Key findings of these studies indicate potential links between FE and shifts in various hepatic pathways, including alterations in the regulation and utilisation of lipids and carbohydrates, as well as features of cellular energy production and expenditure for the modulation of inflammatory and immune-related pathways. Likewise, a study investigating FE-related traits in chickens showed potential effects of selection on immune traits [29]. At the gene expression level in the current study, the differential expression of neuropeptide Y (*NPY*; low > high RFI) and phosphoserine aminotransferase 1 (*PSAT1*; low < high RFI) may contribute to the divergent metabolic profiles associated with FE. NPY is known to influence satiety, energy homeostasis and lipid metabolism [30]. The downregulation of *PSAT1*, which encodes an enzyme involved in serine biosynthesis, may lead to imbalances in amino acid levels within the liver [31]. Moreover, prominent upregulations of *SULT1B* (low > high RFI), affecting thyroid hormone solubility, and *GCHFR* (low > high RFI), involved in the regulation of phenylalanine metabolism in the liver, were observed in the low-RFI group compared to high-RFI birds. The analysis of molecular pathways revealed an enrichment of genes related to cell–substrate adhesion, suggesting liver integrity and health as an important aspect associated with the positive selection of feed-efficient broiler phenotypes. Indeed, it has been reported that less feed-efficient broiler phenotypes appear to be more susceptible to the development of liver pathologies, such as fatty liver haemorrhagic syndrome [32]. In contrast, feed-efficient broiler phenotypes tended to exhibit lower liver weights and reduced hepatic fat accumulation compared to their less-efficient counterparts. This observation is consistent with the findings obtained from the serum metabolome of the current experimental population [14].

### 4.2. Low RFI vs. High RFI Under Restricted Feeding

Under restricted feed supply, at the level of the liver transcriptome, the comparison between birds with high and low RFI showed that genes assigned to immunity and to carbohydrate and lipid metabolism are among the most strongly regulated. *MOGAT1* and *LPIN1* (low < high RFI) emerged as key regulators of triacylglycerol synthesis in response to RFI grouping under feed restriction. This finding aligns with previous observations in fasted mice, where *MOGAT1* expression was altered in the liver [33]. Additionally, genetic variations within *MOGAT1* have been linked to feed conversion efficiency in Hu sheep, suggesting a potential role for the encoded enzyme in nutrient utilisation [34]. For *LPIN1*, a hepatic regulator of lipid metabolism, Kajimoto et al. [35] demonstrated that silencing hepatic *LPIN1* expression in mice led to decreased adipose tissue accumulation and reduced triglyceride levels in the liver and blood. These findings suggest the presence of regulatory pathways that modulate fat storage in response to limited dietary intake, which could be divergently influenced depending on the RFI phenotype. In support of this assumption, it should be noted that the low-RFI birds of the current study, with 77.3 mg/dL, have numerically lower average triglyceride levels in the blood than the high-RFI birds, with 90.6 mg/dL, under restrictive feeding [14]. In high-RFI chickens, the upregulation of *GALK1*, encoding the key enzyme for galactose utilisation, and *SLC2A5*, encoding the fructose transporter, under feed restriction conditions suggests a potential preference for metabolising certain carbohydrates. For *GALK1*, in particular, similar shifts have also been described in response to phytogenic feed additives, accompanied by differences in feed utilisation [6]. The performed enrichment analysis suggested an enhanced activation of the complement cascade in low-RFI birds. Additionally, the enrichment of the heterotypic cell–cell adhesion pathway regulation might be indicative of tissue remodelling processes, encompassing disintegration, regeneration, or maintenance of homeostasis. The influence of the RFI phenotype and dietary regimen on resource allocation between hepatic metabolism and immune function remains an area of investigation [29,36].

### 4.3. Restrictive vs. Ad Libitum Feeding of Low-RFI Phenotypes

The feeding regimen did not result in any differences in body weight in the low RFI, i.e., the high-efficient phenotypes. These results indicate that the birds with low RFI accept a certain range in feed supply for efficient nutrient utilisation. The comparison of hepatic expression profiles between restrictively and ad libitum-fed chickens within the low-RFI phenotype showed a number of DEGs, including *FICD* (FIC domain protein adenylyltransferase) and *CYP2AC2* (cytochrome P450, family 2, subfamily AC, polypeptide 2) among the top regulated transcripts. The increased *FICD* expression in restrictively fed low-RFI broilers might play a regulatory role in hepatic metabolic adaptation to reduce energy expenditure for maintenance. In murine species, *FICD* has been associated with the hepatic modulation of unfolded protein response (UPR) and the liver’s adaptive response to fasting [37,38]. The UPR, as an adaptive organelle stress response mechanism auto-regulated by the accumulation of misfolded proteins in the endoplasmic reticulum’s lumen [39,40], might have been induced by, e.g., glucose deprivation [41]. Implications on organelle function, disease control, and metabolic health need to be addressed [42]. The *CYP2AC2* gene is primarily expressed in the liver and is conserved in the chicken genome, belonging to the cytochrome P450 (CYP) 2AC subfamily [43]. The species-specific function of *CYP2AC2* in *Gallus gallus* remains unclear; however, evidence from other vertebrates, including human and mice [44], has identified the potency of the CYP-dependent roles in fatty acid metabolism, drug metabolism, and xenobiotic pathway activation in the liver’s adaptive response to nutritional and xenobiotic challenges [45]. The observed enrichment of the drug metabolism pathway and associated DEGs in the current study, including *MGST3* and *UDP*-glucuronosyltransferase (Supplementary Table S1; restrictive > ad libitum feeding), further corroborates the liver’s potential to mitigate adverse environmental effects from xenobiotics, drugs, toxins, and other chemicals through intrinsic mechanisms [46]. Thus, the addressed molecular pathways linked FE traits to cellular detoxification and glucuronidation processes, which are crucial for antioxidant defence and redox homeostasis [47,48].

### 4.4. Restrictive vs. Ad Libitum Feeding of High-RFI Phenotypes

The restrictively fed high-RFI phenotype was fed more efficiently than their ad libitum-fed counterparts, although their final body weight was the lowest of all groups. Similar to mammals, where caloric restriction is linked to benefits via enhanced mitochondrial ATP production [49,50], the metabolic response of high-performance broilers might follow comparable principles [19], which might be relevant with regard to body weight and muscle tissue physiology development in other species, including humans. Moreover, it has been reported that various feed restriction regimens primarily affect behaviour, which has an impact on animal welfare [51], especially as animals of commercial broiler genetics exhibit low demand for physical activity [52]. The high-RFI birds appeared to be much more challenged with the restricted feed regimen, which is reflected in the high number of DEGs in this comparison in view of the other contrasts. Specific findings on metabolism are addressed via increased mRNA abundances of *APOA4* (apolipoprotein A4) in feed-restricted chickens compared to ad libitum-fed birds. *APOA4*, an HDL-associated protein, might enhance the efficiency of hepatic-mediated cholesterol transport [53]. The lowered mRNA abundance of *ACAA1* (acetyl-CoA acyltransferase 1) following restricted feeding is indicative of an activated hepatic *PPARα* signalling, as *ACAA1* is operative in the β-oxidation system of the peroxisomes [54]. This indicates an increase in fatty acid degradation and ketone body production in the liver also at the applied moderate feed restriction. The feed restriction prompted lower mRNA abundance of hepatic fibrinogens (*FGG* and *FGB*; Supplementary Table S1) compared to ad libitum-fed broiler chickens. The identified DEGs suggest that the capacity for the regulation of blood coagulation and haemostasis in the high-RFI phenotype is dependent on caloric intake. In contrast, caloric restriction in humans did not show effects on plasma fibrinogen [55]. Kininogen 1 (*KNG1*; Supplementary Table S1), attributed to the ‘blood coagulation’ pathway, was found to be lower expressed in feed-restricted birds compared to ad libitum-fed high-RFI broiler chickens. *KNG1* acts via its splicing variants (i) in blood coagulation, inhibiting the thrombin- and plasmin-induced aggregation of thrombocytes [56], and (ii) is a precursor of the peptide bradykinin, mediating, e.g., vasodilation, natriuresis, diuresis, and decreases the blood glucose level [57]. Fibrinogens have been suggested as biomarkers associated with increased risk for cardiac events in humans [58]. The gene expression pattern could therefore suggest that a controlled and mild feed restriction could lead to health benefits in less feed-efficient broiler chickens, as known from other species. The proportions of white blood cells in restrictively fed broilers showed a significant increase towards higher lymphocytes compared to ad libitum-fed birds [14], which may improve the effective inflammatory response against infections. In this context, research on how changes in the haemostasis and immune system caused by feeding can promote resistance to pathogens is valuable [59]. Therefore, the hepatic RNA profiles indicate a lower susceptibility to blood coagulation as a result of feed restriction in less feed-efficient birds.

## 5. Conclusions

Conclusively, the results show that the expression patterns of the liver, as a post-absorptive tissue, contribute to explaining the phenotypic variation due to RFI in feed-restricted broiler chickens. The lower RFI exhibited by high-efficient broiler chickens is associated with altered expression of genes affiliated with cell–substrate adhesion, lipid metabolism, and fat storage, which is indicative of an effective resource allocation. Restrictive feeding elicited transcriptional changes in the liver, some of which are associated with tissue integrity, possibly contributing to a beneficial liver health status. In addition to the established longitudinal feeding patterns, the results indicate the potential for labour-intensive monitoring of poultry flocks to ensure more individualised feeding, with a focus on subgroups with varying RFI values. In addition to the increased workload and financial costs, this also requires close monitoring through smart farming concepts.

## Figures and Tables

**Table 1:** Average body weight (BW), metabolizable mid BW, and residual feed intake (RFI) values of low- and high-RFI broiler chickens subjected to an ad libitum vs. a restrictive feeding regimen until day 33–37 post-hatch. Data are presented as means with pooled standard error of the mean (SEM).

Trait	Ad Libitum Feeding		Restricted Feeding ^1^			*p*-Value	
	Low RFI (High FE)	High RFI (Low FE)	Low RFI (High FE)	High RFI (Low FE)	SEM	RFI Group	Restriction
*n*	8	8	8	8			
BW (kg)	2.47 ^ab^	2.58 ^a^	2.49 ^ab^	2.26 ^b^	0.12	0.516	0.094
Metabolizable mid BW (g)	188 ^a^	188 ^a^	175 ^b^	170 ^b^	2.95	0.349	<0.001
RFI (g)	−109.1 ^c^	230.2 ^a^	−74.0 ^c^	119.3 ^b^	34.0	<0.001	0.114
FCR (g/g)	1.35 ^b^	1.51 ^a^	1.37 ^b^	1.51 ^a^	0.021	<0.001	0.998

^1^ Feed restriction was intended to account to 90–95% based on results of a reference trial [24]. In this study, feed restriction was 92% in the low-RFI ad libitum group and 80% in the high-RFI ad libitum group. ^a,b,c^ Different superscripts within a row indicate significant differences between groups (*p* ≤ 0.05).

**Table 2:** Pathways and GO terms enriched on the basis of identified DEGs in the liver of low- and high-RFI broiler chickens subjected to an ad libitum vs. a restrictive feeding regimen. Details on affiliated pathways and genes are provided in Supplementary Table S2.

Comparison	Term Name (Database)	Adj. *p*-Value
Low RFI vs. high RFI (ad libitum feeding) ^1^ No. of genes: 328	Positive regulation of cell–substrate adhesion (GO:BP)	0.001
	Positive regulation of cell–matrix adhesion (GO:BP)	0.013
Low RFI vs. high RFI (restricted feeding) No. of genes: 292	Positive regulation of heterotypic cell–cell adhesion (GO:BP)	0.001
	Regulation of heterotypic cell–cell adhesion (GO:BP)	0.016
	Complement cascade (REAC)	0.009
Restrictive vs. ad libitum feeding (low RFI) No. of genes: 140	Drug metabolism—cytochrome P450 (KEGG)	0.022
	Metabolism of xenobiotics by cytochrome P450 (KEGG)	0.024
Restrictive vs. ad libitum feeding (high RFI) No. of genes: 500	Regulation of coagulation (GO:BP)	0.006
	Regulation of response to external stimulus (GO:BP)	0.009
	Regulation of wound healing (GO:BP)	0.014
	Negative regulation of coagulation (GO:BP)	0.028
	Positive regulation of ERK1 and ERK2 cascade (GO:BP)	0.032
	Regulation of blood coagulation (GO:BP)	0.035
	Superoxide metabolic process (GO:BP)	0.042
	Negative regulation of wound healing (GO:BP)	0.042
	Regulation of haemostasis (GO:BP)	0.042

^1^ Low RFI refers to broiler chickens with high feed efficiency (FE); high RFI refers to broiler chickens with low FE; GO—gene ontology; BP—biological pathways; REAC—Reactome Pathway Database; and KEGG—Kyoto Encyclopedia of Genes and Genomes.

## Data Availability

The original contributions presented in the study are included in the article and Supplementary Material; further inquiries can be directed to the corresponding author.

## Supplementary Materials

The following supporting information can be downloaded at: https://www.mdpi.com/article/10.3390/metabo14110625/s1, Table S1: Feed ingredients and analysed chemical composition (as-fed basis) as reported previously [18,23]; Table S2: Gene expression and enrichment analysis in the liver of low and high RFI broiler chickens subjected to an ad libitum vs. a restrictive feeding regimen.
